# Factors Associated with Malnutrition among Under-Five Children: Illustration using Bangladesh Demographic and Health Survey, 2014 Data

**DOI:** 10.3390/children4100088

**Published:** 2017-10-19

**Authors:** Ashis Talukder

**Affiliations:** Statistics Discipline, Khulna University, Khulna 9208, Bangladesh; ashistalukder27@yahoo.com; Tel.: +88-01772063507

**Keywords:** malnutrition, weight-for-age *z*-score, gamma measure, chi-square test, proportional odds model

## Abstract

Child malnutrition remains one of the major public health problems in many parts of the world, especially in a developing country like Bangladesh. Several socioeconomic and demographic factors are responsible for this condition. The present study was conducted to uncover the risk factors associated with malnutrition among under-five children in Bangladesh by analyzing the data from a nationally representative Bangladesh Demographic and Health Survey (BDHS) in 2014. The ordinal dependent variable—child nutrition status (severely malnourished, moderately malnourished, and nourished)—was developed by calculating weight-for-age Z score (WAZ). Bivariate analysis was conducted by performing gamma measure and chi-square test of independence to explore the association between child nutrition status and selected independent variables. To know the adjusted effects of covariates, a popular ordinal model—namely, the proportional odds (PO) model—was considered. All the selected covariates were found highly significant (*p* < 0.01) in the bivariate setup. However, in the multivariate setup, father’s and mother’s education, wealth index, mother’s body mass index (BMI), and antenatal care service during pregnancy were found highly significant (*p* < 0.01) factors for child malnutrition. Among the divisions, only Dhaka had more control on child malnutrition, compared to the Sylhet division. Birth interval of children was also reported as a significant factor at a 5% level of significance. Finally, the results of this paper strongly highlighted the necessity of increasing parent’s education level, improving the mother’s nutritional status, and increasing facilities providing antenatal care service in order to achieve better nutrition status among under-five children in Bangladesh.

## 1. Introduction

Malnutrition among under-five children remains one of the major public health problems in many parts of the world [1]. It is identified as the major cause of death, with an estimate of 45% of all deaths among children aged 0–59 months of age [2]. Worldwide, the prevalence of different forms of malnutrition such as stunting (height-for-age), wasting (weight-for-height), and underweight (weight-for-age) in under-five children were 24.7%, 7.8%, and 15.1%, respectively, in 2014 [3]. Though child malnutrition remains common all over the world, it is most dominant in developing countries [4]. For example, the prevalence of chronic malnutrition was about 39.9% in Africa, and the prevalence rate of underweight was 26.6% in South-East Asia [3]. One study based on the Bangladesh Demographic and Health Survey (BDHS) 2007 reported that the 43% of children in Bangladesh were stunted and 41% were underweight [5], which overflows the threshold of the very high prevalence [6]. Moreover, the wasting rate (17%) was also very alarming for Bangladeshi children [5,6]. Because of various initiatives taken by the Bangladeshi government and different organizations, the rates of stunted (36%), wasted (14%), and underweight (33%) children have been decreasing in recent times [7]. However, it is still not very satisfactory for a developing country like Bangladesh.

In the past, several researchers have tried to determine the actual risk factors for child malnutrition by using various techniques and different statistical models. A popular binary logistic regression model was used in most of the studies [8,9,10,11]. A recent study conducted by Das and Gulshan [12] tried to uncover the risk factors for child malnutrition by applying a binary logistic regression model. However, the nutritional status of children can be classified as severely malnourished, moderately malnourished, and nourished. By considering this ordinal nature of the nutritional status, one can apply ordinal logistic regression models to determine the risk factors for child malnutrition. Very few research works have been done using such ordinal models [13]. Alternatively, a multinomial logistic regression can be applied by ignoring the order information that is present in the response variable. However, applying multinomial logistic regression requires more parameters to estimate and interpret. In this context, an ordinal model—namely, proportional odds (PO) model—can provide a better solution when it satisfies the proportional odds assumption (also known as parallel lines assumption) [14]. Moreover, if there exists a natural ordering, an ordinal analysis can be able to provide different and more powerful results compared to an analysis that ignores ordinality [14]. In this study, an attempt has been made to uncover the associated factors for malnutrition among under-five Bangladeshi children by using BDHS, 2014 data with an application of the PO model.

## 2. Materials and Methods

### 2.1. Data and Variables

This study extracted relevant information from a nationally representative secondary data set (Bangladesh Demographic and Health Survey (BDHS), 2014), which was collected through a joint effort of the National Institute of Population Research and Training (Bangladesh), ICF International (USA), and Mitra and Associates (Bangladesh). For the confirmation of a true representation of the findings at national levels, the analysis of BDHS data was performed by applying sampling weights. Since there was not a self-weighted sample, the estimation procedure utilized weighting factors [15]. Among 17,989 selected households, the effective interviews were done in 98% of all chosen households [7]. In all analyses, sample weights were used for proper standard error and *p*-value estimation to make sample data representative of the entire population.

The BDHS 2014 used anthropometric measurements (weight and height) to investigate the nutrition status of under-five Bangladeshi children. Height-for-age, weight-for-age, and weight-for-height are the widely used indicators for assessing the nutritional health status of a population. Among these three indicators, the anthropometric index weight-for-age can be considered as a good overall index for understanding the nutritional status of children [7]. In addition, weight-for-age is a composite index of height-for-age and weight-for-height [7]. Therefore, this study used only one anthropometric index—weight-for-age—to assess the nutritional status of under-five children in Bangladesh.

On the basis of weight-for-age *Z*-scores (WAZs), the nutritional status of children was divided into three ordinal categories: severely malnourished (<−3.0 WAZ), moderately malnourished (−3.0 to −2.01 WAZ), and nourished (≥−2.0 WAZ). The WAZ were calculated by using the WHO AnthroPlus Software (Geneva, Switzerland) (version 3.2.2, 2011) [16]. The created ordinal variable was considered as the main response variable for final analyses.

A set of socioeconomic and demographic factors related to child malnutrition were considered as covariates to develop the PO model. They were: mother’s education (no or primary, secondary or higher), father’s education (no or primary, secondary or higher), wealth index (categorized based on terciles), mother’s body mass index (BMI) (thin (BMI < 18.5), normal (BMI = 18.5−24.9), over-weight (BMI > 24.9)), place of residence (urban, rural), division (Barisal, Chittagong, Dhaka, Khulna, Rajshahi, Rangpur, Sylhet), antenatal care service during pregnancy (yes, no) and birth interval (<24 months, 24−47 months, ≥48 months).

### 2.2. Statistical Analysis

The association between selected variables and child nutritional status was examined by performing both bivariate and multivariate analyses. Recall that the response variable “child nutritional status” is an ordinal variable. In the bivariate setup, in order to measure the strength of association between the ordinal response and covariates, gamma measure [17] is used when covariates are in ordinal scale, while chi-square is used when they are measured in nominal scale. The estimator of $\hat{\gamma }$ is given by

(1) $$\begin{array}{c} \hat{\gamma }=\frac{C-D}{C+D} \end{array}$$

where *C* is the total number of concordant pairs and *D* is the total number discordant pairs. On the other hand, chi-square has the form

(2) $$\begin{array}{c} \chi^{2}=\sum \frac{{\left(O-E\right)}^{2}}{E} \end{array}$$

where *O* represents the observed frequency. *E* is the expected frequency under the null hypothesis and computed by

(3) $$\begin{array}{c} E=\frac{row\;total\times column\;total}{sample\;size} \end{array}$$

The associated tests use the facts that the statistic $\hat{\gamma }$ follows a normal distribution, with mean = γ and the standard error (SE) calculated from the delta method; and that the statistic chi-square follows a chi-square distribution with (r− 1)(c− 1) degrees of freedom (df), where *r* is the number of categories of the covariate and *c* is the number of categories of the response variable.

In multivariate analysis, the proportional odds (PO) model is considered to assess the adjusted effects of covariates on the nutritional health status of children. Let $Y_{i}\;\left(i=1,2,\cdots ,n\right)$ be the response variable with ordinal categories 1,2,⋯,j,⋯,c. Additionally, let $x_{i}={\left(x_{i1},x_{i2,}\cdots ,x_{ip)}\right)}^{\prime }$ be the vector of *p* covariates related to $Y_{i}.$ The functional form of the proportional odds model which simultaneously considers c−1 cumulative logits can be expressed as

(4) $$\begin{array}{ccc} logit\left[Pr(Y_{i}\leq j)\right] & = & \alpha_{j}+\beta^{\prime }x_{i}\;for\;j=1,\cdots ,c-1, \end{array}$$

where $\beta ={\left(\beta_{1},\beta_{2},\cdots ,\beta_{p}\right)}^{\prime }$ is the vector of regression parameters related with $x_{i}$ and $\alpha_{j}$ represents the intercept for $j^{t}h$ cumulative logit. In (4), $logit\left[P\left(Y_{i}\leq j\right)\right]=log\left[P\left(Y_{i}\leq j\right)/P\left(Y_{i}>j\right)\right]$. Note that in the PO model, the effect of each covariate is assumed to be same for any cumulative logits. Therefore, every time after fitting PO model, we need to check whether the fitted model violates this assumption or not. One can check this assumption by statistical test such as score test [18] or test based on deviance. In this paper, deviance-based test was performed to check the assumption.

## 3. Results

### 3.1. Bivariate Analysis

The results obtained from bivariate analyses are shown in Table 1. From the table it is clear that wealth index, mother’s BMI, and birth interval have a significant monotonic association with the nutritional status of children. Based on gamma estimates, it can be concluded that nutritional status has a significant weak positive relationship with wealth index, mother’s BMI, and birth interval. Therefore, with the increase in wealth index, mother’s BMI, and birth interval, children tended to fall into the “nourished” category of child nutritional status. The percentage of severely malnourished and moderately malnourished children were found higher between the no or primary educated mother (10.9% and 28.7%, respectively), no or primary educated father (10.6% and 27.8%, respectively), rural area (8.3% and 26.1%, respectively), Sylhet division (10.5% and 28.5%, respectively), and those who did not receive any antenatal care (ANC) service during pregnancy (13.2% and 25.4%, respectively). All these selected covariates were found significantly related to the nutritional health status of children (*p* < 0.001).

### 3.2. Regression Analysis

The effects of the selected covariates were estimated by fitting the proportional odds (PO) model. Note that the deviance-based chi-square test provided evidence that the data satisfied the parallel lines assumption ($\chi^{2}$ statistic = 23.338, df = 16, *p*-value = 0.105), which indicates that for each of the chosen covariates a single parameter can be used to model separate logits of cumulative probabilities. The estimated effects are displayed in Table 2. From the table it is observed that the odds of staying in a worse state (severely malnourished and moderately malnourished) of nutritional status was [1 − exp (0.776)] = 22% lower for the children having secondary or higher educated mother (*p* = 0.001), compared to no or primary educated mother. These odds were 24% lower for the children having a secondary or higher educated father (odds ratio= 0.760, *p* = 0.003). Compared to the children from poor income families, the odds of switching status from malnourished to nourished nutritional status was 24% and 43% lower for the children from middle class families (*p* = 0.001) and rich families (*p* < 0.001), respectively. Among the division, the risk of having malnourished children was relatively lower in Dhaka (odds ratio = 0.882, *p* < 0.05), compared to Sylhet. Children with normal weighted mother had 43% lower odds of staying in the worst state of nutritional status. It was also observed that the children with overweighted mother had a lower risk of having a poor nutritional status. The mothers receiving antenatal care service during pregnancy had a lower risk of having a malnourished child (odds ratio = 0.777, *p* = 0.003). Moreover, the children having birth interval 24−47 months had less chance of staying in the worst state of nutritional status (odds ratio = 0.843, *p* < 0.05) compared with the children having less than 24 months birth interval. However, place of residence had no significant effect on the nutritional status of children.

## 4. Discussion

The findings of this study provide evidence that around 7.9% and 24.5% of sampled children were severely and moderately malnourished, respectively, which is slightly lower than the last few decades. This is due to several interventions taken by the Bangladeshi government and various development organizations. This study states the areas of future improvement of the nutritional status of under-five children in Bangladesh.

In this study, both bivariate and multivariate analyses were performed. From the bivariate analysis, it was found that mother’s education, father’s education, wealth index, mother’s BMI, place of residence, division, antenatal care service during pregnancy, and birth interval were significantly associated with the nutritional status of children. On the other hand, a PO model was used to estimate the adjusted effects of selected covariates by considering the ordinal nature of the dependent variable.

The results of the PO model showed that both father’s and mother’s education had significant positive effects on child malnutrition. Children with secondary or higher educated father (or mother) were around 20–25% less likely to stay in the worst nutrition condition, compared to the children with illiterate fathers (or mothers). This finding coincides with several previous studies [19,20,21,22,23,24]. This is realistic because the higher-educated mother has better knowledge of child health and nutrition. Moreover, the higher-educated father also has significant contribution to family income as well as choosing healthy foods for his family. The prevalence of child malnutrition is relatively higher for the children belonging to the poor category of the wealth index. Apart from their limited wealth, the lesser education of poor parents resulting in a lack of knowledge about food and nutrition may be the main cause for increasing rates of malnourished children among poor families in Bangladesh. Therefore, the policymakers may take initiatives to increase not only education level, but also consciousness about child nutrition among poor people. In this way, they can be educated by giving proper guidance about buying nutritious food by spending money within their limit.

In addition, mother’s BMI had a significant negative effect on child malnutrition, indicating that healthier mothers have less risk of having malnourished children. The mothers receiving antenatal care service during pregnancy had lower odds of staying in the worst state of child nutritional status, compared to the mothers not receiving the service. Both of these suggest that the good nutritional status of the mother should be ensured in pregnancy in order to reduce child malnutrition. The policy maker should keep in mind that good nutritional status of the mother can confirm better breastfeeding and help to quickly recover from both physical and mental stress during pregnancy.

Finally, the PO model indicated that children with higher birth interval had a lower risk of staying in a poor nutritional state. Moreover, the Dhaka division had more control over child malnutrition than the Sylhet division. This may be due to the implementation of various intervention programs in the capital city of Bangladesh. Therefore, I strongly suggest that the policymaker implement more programs associated with child health to improve child nutritional status in every division of Bangladesh.

## 5. Conclusions

The prevalence of child malnutrition in Bangladesh is still high. Potential factors associated with child malnutrition are several, including parents’ education level, wealth index, mother’s BMI, division, antenatal care service during pregnancy, and birth interval of children. The results of this paper strongly highlight the necessity of increasing parents’ education level, improving the mother’s nutritional status, and increasing antenatal care service facilities during pregnancy in order to achieve better nutritional status among under-five children in Bangladesh.

## Figures and Tables

**Table 1:** Assessing the association between selected covariates and nutrition status of under-five children using gamma measure and chi-square test.

Covariates	Measurement Scale	Nutrition Status			$\hat{\gamma }$ (*p*-Value)	$\chi^{2}$ (*p*-Value)
		Severely Malnourished n (%)	Moderately Malnourished n (%)	Nourished n (%)		
Mother’s education	Nominal	-	-	-	-	-
No or primary		345 (10.9)	913 (28.7)	1920 (60.4)	-	149.499
Secondary or higher		220 (5.5)	845 (21.2)	2930 (73.3)		(0.000)
Father’s education	Nominal	-	-	-	-	-
No or primary		424 (10.6)	1116 (27.8)	2470 (61.6)	-	173.210
Secondary or higher		141 (4.5)	642 (20.3)	2378 (75.2)		(0.000)
Wealth index	Ordinal	-	-	-	-	-
Poor		288 (11.8)	741 (30.5)	1403 (57.7)	0.300	
Middle		172 (7.1)	629 (25.9)	1627 (67.0)	(0.000)	
Rich		105 (4.5)	388 (16.8)	1820 (78.7)		
Place of residence	Nominal	-	-	-	-	-
Rural		448 (8.3)	1403 (26.1)	3515 (65.5)		42.763
Urban		117 (6.5)	356 (19.7)	1334 (73.8)		(0.000)
Mother’s BMI	Ordinal	-	-	-	-	-
Thin		211 (13.3)	502 (31.6)	876 (55.1)	0.323	
Normal		303 (7.2)	1035 (24.6)	2868 (68.2)	(0.000)	
Overweight		48 (3.6)	209 (15.5)	1095 (81.0)		
Division	Nominal	-	-	-	-	-
Dhaka		176 (7.0)	540 (21.5)	1801 (71.6)	-	63.571
Barisal		31 (7.5)	115 (28.0)	265 (64.5)		(0.000)
Chittagong		132 (8.7)	406 (26.8)	978 (64.5)		
Khulna		29 (5.3)	112 (20.5)	405 (74.2)		
Rajshahi		54 (7.2)	189 (25.0)	512 (67.8)		
Rangpur		62 (8.5)	203 (27.9)	463 (63.6)		
Sylhet		73 (10.5)	199 (28.5)	425 (61.0)		
ANC service	Nominal	-	-	-	-	-
Yes		202 (6.1)	667 (20.1)	2442 (73.8)	-	70.150
No		116 (13.2)	223 (25.4)	539 (61.4)		(0.000)
Birth interval (months)	Ordinal	-	-	-	-	-
<24		393 (8.6)	1192 (26.2)	2961 (65.1)	0.126	-
24–47		109 (6.2)	376 (21.4)	1274 (72.4)	(0.000)	
≥48		56 (7.0)	180 (22.6)	561 (70.4)		

ANC: antenatal care.

**Table 2:** Proportional odds model based estimated effects of selected covariates.

Covariate	Estimate	Odds Ratio (95% CI)	*p*-Value
Intercept ($\alpha_{1}$)	−0.872	-	0.000
Intercept ($\alpha_{2}$)	0.809	-	0.000
Mother’s education			
No or primary (ref)			
Secondary or higher	−0.253	0.776 (0.651, 0.906)	0.001
Father’s education			
No or primary (ref)			
Secondary or higher	−0.274	0.760 (0.653, 0.918)	0.003
Wealth index			
Poor (ref)			
Middle	−0.278	0.757 (0.636, 0.899)	0.001
Rich	−0.557	0.572 (0.456, 0.717)	0.000
Place of residence			
Urban (ref)			
Rural	0.013	1.013 (0.854, 1.202)	0.879
Mother’s BMI			
Thin (ref)			
Normal	−0.554	0.574 (0.491, 0.671)	0.000
Overweight	−0.924	0.396 (0.308, 0.509)	0.000
Division			
Sylhet (ref)			
Dhaka	−0.125	0.882 (0.657, 0.968)	0.040
Barisal	0.197	1.217 (0.942, 1.575)	0.130
Chittagong	−0.152	0.858 (0.654, 1.125)	0.269
Khulna	−0.020	0.980 (0.738, 1.299)	0.885
Rajshahi	0.115	1.122 (0.852, 1.477)	0.409
Rangpur	0.098	1.103 (0.844, 1.441)	0.471
ANC service			
No (ref)			
Yes	−0.252	0.777 (0.654, 0.922)	0.003
Birth interval (months)			
<24 (ref)			
24–47	−0.170	0.843 (0.711, 0.995)	0.044
≥48	−0.133	0.875 (0.693, 1.102)	0.257

CI: Confidence interval.
